# The Future Is Youth's Freehold

**Published:** 1916-11-18

**Authors:** 


### THE HOSPITALS OF THE UNITED STATES.

### The Future is Youth's Freehold.

Looking back thirty years and more, and recalling the condition of hospitals and organisations for the relief of sickness and the treatment of disease in the United States, the visitor of to-day is filled with rejoicing by the vast improvement in administration, in construction, in equipment, in scientific development, in the large-minded working out of new methods, and in the search for and adoption of high standards. The splendid technique and method and the ability and keenness of the medical superintendents, with the splendid moral they have secured, are notable features of the hospital system of the United States of to-day.

Again, the successful efforts which have been made to raise the standard of medical practice, the efficiency and scope of the plans and schemes adopted and in preparation to extend and improve the quality of medical education, the work of the professors and teachers and the students for whom they are responsible, have been very remarkable in the United States during the last thirty and especially during the last twenty, years.

The proceedings at the sessions of the American Hospital Association in Philadelphia revealed the value and extent of these vast improvements and changes. It used to be said that doctors, like parsons, were seldom men of business, and the institution of the panel system in Great Britain, with its lamentable effects upon a large number of medical practitioners, called forth the criticism that the profession was degenerating from a learned profession to a mere trade organisation. The medical profession in the United States is at present happily free from the panel system, though strenuous efforts are being made in several States to introduce a system of compulsory State insurance for all.

This American Hospital Association is comparatively young, and it has taken fifteen years of self-denying effort on the part of the more active hospital spirits throughout the United States to bring it up to its present standard, which may be expected to have important results for good upon the majority, at least, of the hospitals which have already identified themselves with it. We are encouraged to make this affirmation as the result of a close study of the men present and the methods pursued at the Philadelphia Conference. There was, from the outset, evidence that the many hundreds of members present were united in a resolve to obtain a new constitution and to secure its unanimous adoption this year. At one time such a result seemed doubtful and even impossible. But the ability of the President, Dr. Winford H. Smith, Superintendent of Johns Hopkins Hospital, Baltimore; Dr. H. P. Howard, Superintendent, Peter Bent Brigham Hospital, Boston, Mass.; Dr. H. M. Hurd, and Mr. Richard P. Borden, Trustee of the Union Hospital, Fall River, Mass., with others, who exhibited sound judgment and infinite patience, resulted in the new constitution and by-laws being dealt with in detail and fully considered, and the whole of them were adopted at great meetings with unanimity and without a single division.

The truth is that there is to-day a unanimous desire throughout the members of the Association to support the highest standards of efficiency everywhere. The general wish evidently is, through the proceedings and action of the American Hospital Association, to raise the standard of efficiency and method to the highest possible point throughout the hospitals of the United States.

We welcome, further, the recognition by the leaders of this Association of the importance of making each Annual Conference exercise an important educational influence upon the members and upon all associated with them in the administration of the hospitals with which they are connected. We feel some confidence that the improvements effected in this direction under the new Council just appointed will render the proceedings at the next and future Conferences of general interest and supreme importance.

It was not possible to attempt anything really practical in these directions until the new constitution and by-laws had been accepted by the members. Now that this great change for the better has been accomplished, the field of progress lies open for cultivation to the trustees and officers elected at Philadelphia on September 29, 1916. Heretofore the programme for each session of these Conferences has been far too full; there has been an absence of classification of subjects and of selection of those which possess urgency or high educational value. A crowded programme containing papers of very varying value and dealing with many different departments or subjects tends to confuse the members. It tends to tire those who are the most attentive, and to occupy so large an amount of time that discussion of a practical and extended nature, which under proper control is the high-road to high educational development in sessions of this kind, has been impossible at recent meetings.

The newspaper press was well represented, but the reporters were left without direction or the necessary help to enable them to handle effectively the subjects dealt with. This resulted in the briefest possible reports, not one of which contained a clear exposition of some really important questions dealt with. A competent committee, charged with the duty of arranging a programme for every session, could readily have selected the most interesting subject and placed it first on the programme, with time enough to enable it to be discussed by a few of the ablest members, selected because of their authority in relation to the questions dealt with. The results of such a rearrangement of the business at future Conferences would be of great benefit. Synopses of all these papers might have been prepared and printed, which could have been handed to the reporters with an indication of the relative value of each and the chief points of interest. It was difficult enough for the best informed members of each audience to follow the sessional work at Philadelphia, and it must have been impossible for an ordinary reporter to deal with much of it in the circumstances. This probably accounts for the inadequacy of the reports and their omission of much which, under a different system, could have been presented in a form calculated to arouse the interest of newspaper readers and to bring many new recruits of value to the support of hospitals throughout the country.

The President, Dr. Winford Smith, did yeoman service under immense difficulties, which he surmounted with a courage and skill proclaiming him to be a man in the first rank of hospital administrators. He was ably assisted by the Treasurer, Mr. Asa S. Bacon, of the Presbyterian Hospital, Chicago, who was courtesy itself, and by the three Vice-Presidents, as well as by the Executive Committee, of which Dr. Frederick A. Washburn is the Chairman, and their officers. To them the unanimous and grateful thanks of the Conference were extended for their valuable services, services which resulted in making much of the business done of general interest and importance.

The proceedings at the later sessions of the Conference were full of interest, and exhibited the large numbers of men of progress to be found in the ranks of the younger superintendents of both sexes who were present and took a useful part in the proceedings. This is as it should be, for the future is youth's freehold, and the United States, as one of the younger nations, may one day lead the world in science, hospital administration, and the treatment of disease and accidents. If so, there is already evidence that it will be under a system of hospitals which will provide for the care and cure of members of all classes of the population, from the highest and richest to the poorest and most helpless, in the day of physical disablement in any form.

